# Sex-Based Differences in Factors Associated With Sugar-Sweetened Beverage Consumption Among Korean High School Students

**DOI:** 10.3389/fnut.2022.907922

**Published:** 2022-06-14

**Authors:** Jin Suk Ra, Moonkyoung Park

**Affiliations:** College of Nursing, Chungnam National University, Daejeon, South Korea

**Keywords:** adolescent, sugar-sweetened beverages, sex, dietary behavior, health habit

## Abstract

This study aimed to identify sex-based differences in the individual and environmental factors associated with sugar-sweetened beverage (SSB) consumption among Korean high school students. Secondary data were obtained from the 15th (2019) Korea Youth Risk Behavior Web-based Survey. In this study, we analyzed data from 13,066 high school students (5,874 boys and 7,192 girls) who answered questions regarding SSB consumption and individual and environmental factors. Complex sampling analysis (descriptive statistics and logistic regression analysis) was conducted using the SPSS Statistics 26.0 software. Most adolescents (97% boys and 95.2% girls) reported having consumed SSBs in the last seven days. Individual factors, such as increased stress, sleep dissatisfaction, and fast-food intake (more than thrice a week) were positively associated with SSB consumption among adolescent boys and girls. Environmental factors like high education levels (above college) of mothers were negatively associated with SSB consumption among both boys and girls. Furthermore, current alcohol consumption, smoking, low vegetable intake (less than thrice a week) in boys, and more than 2 h a day of screen-based sedentary behavior in girls were positively associated with SSB consumption. According to the results, individual factors associated with SSB consumption varied according to the sex of adolescents. Thus, sex differences in factors associated with SSB consumption in adolescents should be considered as basic knowledge for developing strategies for reducing SSB consumption.

## Introduction

The phrase “sugar-sweetened beverages (SSBs)” refers to sugar-sweetened and artificially sweetened beverages, such as soda, fruit-flavored drinks, and sports or energy drinks (1, 2). Owing to the added sugar, SSB consumption is associated with increased calorie intake, resulting in excessive weight gain or obesity (1, 3). In addition, SSB consumption has adverse effects on metabolic (e.g., type 2 diabetes and dyslipidemia) and dental health (e.g., dental caries) among children and adolescents (4). It is also associated with mental health conditions and behavioral problems such as hyperactivity, depression, and anxiety among children and adolescents (5–7).

Adolescents, especially high school students, are the leading consumers of SSBs in the USA and Korea (8, 9). A recent study by Southerland et al. (10) showed that approximately 63% of adolescents in the USA consumed SSBs at least once a day; the SSBs accounted for more than 9% of their daily calorie consumption. Kim et al. (11) reported that approximately 94% of Korean high school students consumed SSBs at least once a week, and approximately 40% of them consumed SSBs more than once a day. Furthermore, the daily average sugar consumption among adolescents was approximately 13% higher than the daily average sugar consumption among other age groups (8). Thus, adolescents, especially high school students, may experience significantly adverse effects of SSBs. In this context, Joo et al. (8) highlighted the need for interventions to reduce SSB consumption among high school students. Researchers have also emphasized the importance of identifying significant factors associated with SSB consumption among adolescents, especially high school students, for the development of effective interventions (12, 13).

Although the evidence regarding factors associated with SSB consumption among children and adolescents majorly comes from Western countries, it should be noted that lifestyle patterns (e.g., dietary behaviors) are influenced by individual, sociocultural, and environmental factors (1, 13). Furthermore, factors associated with SSB consumption may differ according to race, ethnicity, and sex (2, 14). Thus, the factors associated with SSB consumption for Korean high school students may vary compared to those for students in Western countries. Furthermore, there may be sex differences in the factors associated with SSB consumption among Korean adolescents.

Based on socioecological models of health-related behaviors, Watts et al. (13) emphasized the need to focus on the multiple (individual and environmental) factors associated with SSB consumption. Previous studies have indicated several potential individual factors associated with SSB consumption among adolescents, including sex, age, screen-based sedentary behavior, physical activity, fruit and vegetable intake, fast-food intake, sleep satisfaction, depressive symptoms, stress, current alcohol consumption, and current smoking behaviors (1, 2, 12, 13). In addition, environmental factors such as the socioeconomic status of the family, educational level of parents, and area of residence were potentially associated with SSB consumption among adolescents (1, 2). Thus, this study aimed to identify sex differences in individual and environmental factors associated with SSB consumption among Korean high school students.

## Materials and Methods

### Research Design and Sample

**Figure 1:**
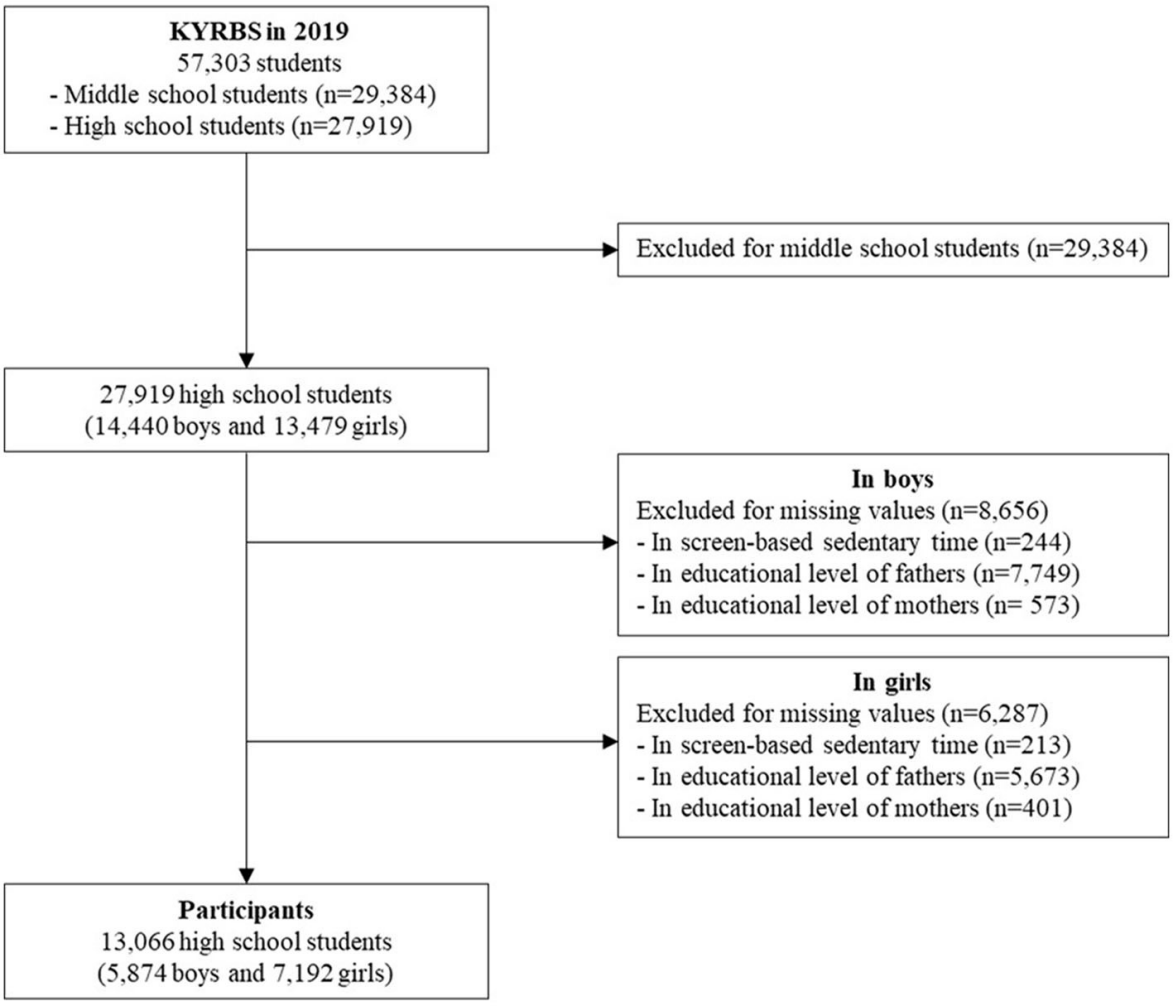
Sampling process. KYRBS, Korea Youth Risk Behavior Web-based Survey.

In this cross-sectional study, we analyzed secondary data obtained from the 15th (2019) Korea Youth Risk Behavior Web-based Survey (KYRBS). KYRBS is an annually-conducted anonymous self-report online survey for middle and high school students to identify the health behavior of Korean adolescents (15). The survey covered 60,100 middle and high school students from 800 schools across 17 provinces of South Korea. Of the 60,100 adolescents, 57,303 adolescents (95.3%), including 29,384 middle school students and 27, 919 high school students, responded to the survey. After excluding all middle school students and 14,943 high school students with missing values, we analyzed data from 13,066 high school students (5,874 boys and 7,192 girls) who answered questions regarding SSB consumption and individual and environmental factors (Figure 1).

### Measurement

The questions and response categories for the individual and environmental factors have been listed and described in Table 1. Sodas, energy drinks, and other sweetened drinks with added sugar (e.g., fruit-flavored and sports drinks) were considered as SSBs in this study. In addition, SSB consumption was assessed using three items asking about the consumption days of each soda, energy drink, and other sweetened drinks, respectively, in the last seven days. In the one or more items assessing the days of SSB consumption, more than 1 day of SSB consumption in the last seven days was considered as indicative of consuming SSBs, following the recommendation of the American Academy of Pediatrics to remove all SSBs from the diets of children and adolescents (16).

**Table 1:** Measurement of individual and environmental factors.

**Variables**	**Measurement**
**Individual factors**	
Grade	1st, 2nd, and 3rd
Depressive symptoms	Single question to assess the sense of sadness or hopelessness in the last 12 months. Available responses of yes/no
Stress	Single question to subjectively assess the stress levels. Available responses classified into yes (extremely high, high, and low)/no (almost none and not at all)
Current alcohol consumption	Single question to assess alcohol consumption in the last 30 days. Available responses classified into yes (frequency more than 1–2 days/no (none)
Current smoking consumption	Single question to assess smoking consumption in the last 30 days. Available responses classified into yes (frequency more than 1–2 days)/no (none)
Moderate and vigorous physical activity	Two questions to assess number of days moderate and vigorous physical activity undertaken in the last 7 days. Available responses classified into <3 days/more than 3 days following the physical activity guide for Korean in 2013
Screen-based sedentary time	Single question to assess the average number of hours, or minutes, per day spent on screen-based sedentary activities, including watching TV, playing video games, using the internet (except for the purpose of learning). Available responses classified into <2 h/2 h or more per day
Sleep satisfaction	Single question to assess sleep satisfaction in the last 7 days. Available responses classified into dissatisfied (very dissatisfied and dissatisfied)/satisfied (very satisfied and satisfied)
Fruit intake	Single question to assess fruit (except fruit juice) intake in the last 7 days. Available responses classified into <3 days/more than 3 days following classification proposed by a previous study with adolescents (1, 11)
Vegetable intake	Single question to assess vegetable (except Kimchi) intake in the last 7 days. Available responses classified into <3 days/more than 3 days following classification proposed by a previous study with adolescents (1, 11)
Fast-food intake	Single question to assess fast-food intake in the last 7 days. Available responses classified into less than 3 days/more than 3 days following classification proposed by a previous study with adolescents (11)
**Environmental factors**	
Socioeconomic status of family	Single question to assess the household's perceived economic status. Available responses classified into high, middle, and low
Area of residential location	Single question to assess the size of the residential location. Available responses classified into metropolis, mid-sized city, and rural area
Educational level of fathers	Single question to assess the educational level of fathers. Available responses classified into below middle school, high school, and above college
Educational level of mothers	Single question to assess the educational level of mothers. Available responses classified into below middle school, high school, and above college

### Ethical Considerations

This current study was exempted from the Institutional Review Board's (IRB) review, as it was conducted using secondary data from the 15th KYRBS, 2019 (Approval no. 202109-SB-207-01).

### Statistical Analysis

Complex sampling analysis was conducted using the SPSS software (version 26.0; IBM, Armonk, NY, USA), adhering to the guidelines proposed by the KYRBS, 2019. The SSB consumption and individual and environmental factors were analyzed using descriptive statistics in the form of frequency and percentage. In addition, logistic regression analysis was performed to identify the sex differences in factors associated with SSB consumption among high school students. To account for the effects of the additional variables, all individual and environmental factors were factored into the logistic model, and an eventually adjusted odds ratio (AOR) was calculated.

## Results

### Descriptive Analysis of SSB Consumption and Individual and Environmental Factors Among South Korean High School Students

As shown in Table 2, 5,700 boys (97% of the 5,874 boys included in the study) and 6,836 girls (95.2% of the 7,192 girls included in the study) reported having consumed SSBs in the last seven days. Furthermore, the sex-wise distribution of the individual and environmental factors associated with SSB consumption has been presented in Table 2.

**Table 2:** Characteristics of sugar-sweetened beverages and individual and environmental factors among participants.

**Variables**	**Categories**	**Boys (*n* = 5,874)**	**Girl (*n* = 7,192)**
		***n*** ^*^(%)^†^	***n*** ^*^(%)^†^
Sugar-sweetened beverages consumption experience (in last 7 days)	No	174 (3.0)	356 (4.8)
	Yes	5,700 (97.0)	6,836 (95.2)
Kinds of sugar-sweetened beverages^††^	Soda	5,089 (86.2)	5,288 (73.9)
	Energy drink	2,094 (36.4)	2,247 (31.8)
	Other sweet drink (e.g., sports and fruits flavored drinks)	5,340 (89.7)	6,278 (87.4)
**Individual factors**			
Grade	1st	1,996 (33.5)	2,442 (33.6)
	2nd	1,894 (31.7)	2,324 (31.1)
	3rd	1,984 (34.8)	2,426 (35.3)
Depressive symptoms	Yes	1,435 (24.8)	2,518 (35.0)
	No	4,439 (75.2)	4,674 (65.0)
Stress	Yes	4,589 (78.5)	6,467 (89.9)
	No	1,285 (21.5)	725 (10.1)
Current alcohol consumption	Yes	1,501 (25.5)	1,311 (17.9)
	No	4,373 (74.5)	5,881 (82.1)
Current smoking consumption	Yes	805 (13.8)	325 (4.5)
	No	5,069 (86.2)	6,867 (95.5)
Moderate and vigorous physical activity (a week)	≥3 days	2,945 (51.0)	1,364 (18.7)
	<3 days	2,929 (49.0)	5,828 (81.3)
Screen-based sedentary time (a day)	≥2 h	4,230 (72.2)	5,282 (73.5)
	<2 h	1,644 (27.8)	1,910 (26.5)
Sleep satisfaction	Dissatisfied	4,754 (81.0)	6,388 (88.9)
	Satisfied	1,120 (19.0)	804 (11.1)
Fruit intake (a week)	<3 times	2,605 (44.5)	3,031 (42.1)
	≥3 times	3,269 (55.5)	4,161 (57.9)
Vegetable intake (a week)	<3 times	5,163 (88.0)	6,545 (91.3)
	≥3 times	711 (12.0)	647 (8.7)
Fast-food intake (a week)	<3 times	4,298 (72.2)	5,586 (77.5)
	≥3 times	1,576 (27.8)	1,606 (22.5)
**Environmental factors**		
Socioeconomic status of family	High	2,376 (40.6)	2,570 (35.9)
	Middle	2,719 (46.5)	3,666 (51.1)
	Low	779 (12.9)	956 (12.9)
Area of residential area	Metropolis	3,105 (51.4)	3,761 (51.4)
	Mid-sized cities	2,438 (44.0)	2,947 (43.7)
	Rural area	331 (4.6)	484 (4.9)
Educational level of fathers	Above college	3,730 (64.2)	4,658 (65.4)
	High school	1,984 (33.1)	2,379 (32.7)
	Below middle school	160 (2.6)	155 (2.0)
Educational level of mothers	Above college	3,491 (59.5)	4,318 (60.3)
	High school	2,271 (38.7)	2,747 (38.0)
	Below middle school	112 (1.8)	127 (1.7)

* *Unweighted;*

† *Weighted;*

†† *Multiple response*.

### Association Between Individual and Environmental Factors and SSB Consumption Among South Korean High School Students

The individual and environmental factors associated with SSB consumption are shown in Table 3. For both boys and girls, individual factors like stress (AOR = 1.75, *p* = 0.001 among boys; AOR = 1.34, *p* = 0.020 among girls) and sleep dissatisfaction (AOR = 1.39, *p* = 0.028 among boys; AOR = 1.54, *p* = 0.011 among girls) were positively associated with SSB consumption. In addition, fast-food consumption more than thrice a week was significantly and positively associated with SSB consumption (AOR = 4.44, *p* < 0.001 among boys; AOR = 4.51, *p* < 0.001 among girls). Finally, environmental factors like the educational level of mothers (above college) were negatively associated with SSB consumption among both boys (AOR = 0.36, *p* = 0.025) and girls (AOR = 0.31, *p* = 0.028).

**Table 3:** Individual and environmental factors associated with sugar-sweetened beverage consumption among high school students.

**Variables**	**Categories**	**Boys (*****n*** **= 5,874)**			**Girls (*****n*** **= 7,192)**		
		**AOR**	**95% CI**	***p***	**AOR**	**95% CI**	***p***
**Individual factors**							
Grade	3rd	1.25	0.87–1.79	0.219	0.81	0.62–1.07	0.137
	2nd	1.18	0.84–1.64	0.337	1.21	0.86–1.70	0.267
	1st (Ref.)	1			1		
Depressive symptoms	Yes	1.00	0.67–1.48	0.997	1.04	0.82–1.30	0.756
	No (Ref.)	1			1		
Stress	Yes	1.75	1.27–2.41	0.001	1.34	1.04–1.72	0.020
	No (Ref.)	1			1		
Current alcohol consumption	Yes	2.26	1.42–3.60	0.001	1.11	0.77–1.58	0.580
	No (Ref.)	1			1		
Current smoking consumption	Yes	2.24	1.08–4.64	0.030	1.16	0.60-2.25	0.654
	No (Ref.)	1			1		
Moderate and vigorous physical activity (a week)	≥3 days	1.31	0.97–1.77	0.081	0.80	0.62–1.05	0.104
	<3 days (Ref.)	1			1		
Screen-based sedentary time (a day)	≥2 h	0.90	0.65–1.24	0.503	1.41	1.05–1.71	0.019
	<2 h (Ref.)	1			1		
Sleep satisfaction	Dissatisfied	1.39	1.04–1.29	0.028	1.54	1.10–2.16	0.011
	Satisfied (Ref.)	1			1		
Fruit intake (a week)	<3 times	1.13	0.78–1.65	0.515	1.16	0.92–1.47	0.220
	≥3 times (Ref.)	1			1		
Vegetable intake (a week)	<3 times	2.56	1.78–3.70	<0.001	1.25	0.86–1.80	0.241
	≥3 times (Ref.)	1			1		
Fast-food intake (a week)	≥3 times	4.44	2.55–7.73	<0.001	4.51	3.03–6.71	<0.001
	<3 times (Ref.)	1			1		
**Environmental factors**							
Socioeconomic status of family	High	1.18	0.76–1.85	0.456	1.07	0.73-1.57	0.723
	Middle	1.21	0.78–1.87	0.389	0.94	0.63–1.36	0.722
	Low (Ref.)	1			1		
Area of residential location	Metropolis	0.91	0.42–1.93	0.781	0.85	0.59-1.23	0.380
	Mid-sized cities	0.60	0.28-1.29	0.191	1.04	0.70–1.48	0.941
	Rural area (Ref.)	1			1		
Educational level of fathers	Above college	1.26	0.46–3.49	0.652	0.93	0.38–2.25	0.864
	High school	1.18	0.81–1.72	0.390	1.27	0.95–1.69	0.103
	Below middle school (Ref.)	1			1		
Educational level of mothers	Above college	0.36	0.15–0.88	0.025	0.31	0.11–0.88	0.028
	High school	0.99	0.69–1.47	0.911	0.86	0.66–1.12	0.268
	Below middle school (Ref.)	1			1		

*AOR, Adjusted odds ratio; 95% CI, 95% confidence interval; Ref., Reference*.

Current alcohol consumption (AOR = 2.26, *p* = 0.001), current smoking behaviors (AOR = 2.24, *p* = 0.030), and vegetable intake of less than thrice a week (AOR = 2.56, *p* < 0.001) were positively associated with SSB consumption among boys. More than 2 h of screen-based sedentary behavior per day (AOR = 1.41, *p* = 0.019) was positively associated with SSB consumption among girls.

## Discussion

Using national data, this study identified sex-based differences in factors associated with SSB consumption among Korean high school students. According to the results, almost all adolescents—boys and girls—had consumed SSBs in the last seven days. These results indicate that SSB consumption may be a common and routine dietary behavior, which is likely to progress to habitual behavior. A previous study also reported that adolescents habitually tend to consume SSBs (17). The World Health Organization (WHO) noted that SSB consumption was detrimental to dietary habits owing to increased intake of free sugar, which is a risk factor associated with non-communicable diseases like obesity (18). Accompanied by increasing autonomy and self-regulation, adolescence is a critical developmental period for good habit formation; these habits may last for life (19). Thus, the development of various interventions focused on decreasing SSB consumption is important to promote good health in adolescence and throughout life. In this context, it is essential to identify the factors associated with SSB consumption among adolescents.

In our study, increased stress, sleep dissatisfaction, and frequent fast-food intake were significantly associated with increased SSB consumption for both boys and girls. In addition, high educational qualifications of mothers (above college level) were associated with decreased SSB consumption for both boys and girls. According to Adam and Epel (20), stress can induce cortisol secretion via stimulation of the hypothalamic–pituitary–adrenal (HPA) axis. Furthermore, low-intensity and chronic stress can also cause “stress-induced food reward dependence,” which increases appetite and food intake, leading to unbalanced dietary intake (20). Individuals experiencing stress tend to prefer a sweet taste (21). Kim et al. (11) reported that individuals experiencing stress may consume 1.12 to 1.39 times the amount of SSBs at least once a day as compared to individuals who are not currently experiencing stress, regardless of sex. Stress management has been effective in decreasing SSB consumption (22). Thus, even though physical activity was not associated with SSB consumption in adolescents in this study, stress-relieving interventions (e.g., physical activity) could be effective in reducing stress-induced SSB consumption. The results of a meta-analysis of the effects of stress reduction interventions in adolescents have indicated that physical activity is more effective for stress relief than counseling and education (23).

In line with the results of this study, previous studies have shown that short sleep duration results in increased consumption of SSBs, such as energy drinks (24). In a study conducted with adolescents in the USA, a sleep duration of <8 h was associated with a 1.4–1.5-fold increase in SSB consumption (excluding energy drinks) and a 1.1–2.3-fold increase in energy drink consumption to prevent sleepiness (9, 24). Similarly, average sleep duration of 6.5 h for five nights significantly increased carbohydrate- and sugar intake among adolescents as compared to an average sleep duration of 9.5 h for five nights (25). These results may be associated with increased neuronal activation in response to eating food (including calorie-dense foods like SSBs and snacks with added sugar) following sleep deprivation (26). Thus, Duraccio et al. (25) supposed that sleep dissatisfaction may foster unhealthy dietary habits, facilitating sugar intake. Korean high school students tend to have longer wake duration (with an average sleep duration of 6 h 3 min per day) (27), owing to excessive competition for college entrance exams (28). Thus, they may experience sleep dissatisfaction and day time sleepiness, which may in turn facilitate SSB consumption. Thus, there is a need to develop strategies for attaining optimal sleep satisfaction to decrease SSB consumption.

In line with the results of this study, adolescents in the USA who visited fast-food restaurants three or more times a week consumed approximately three times more SSBs compared to adolescents who visited fast-food restaurants less than thrice a week (9). Kim and Lee (29) reported that frequent fast-food intake was associated with increased SSB consumption. Similarly, the overall SSB consumption among children and adolescents in the USA decreased with the development of sugar-reduced menus in fast-food restaurants (30). High school students in Korea spend most of their time at school; they may consume increasing amounts of SSBs—along with fast food (e.g., hamburgers and pizzas)—that are readily available at cafeterias in or near their school (11). According to previous studies, adolescents who regularly visited the school cafeteria tended to consume more SSBs (31, 32). Thus, a school environment that encourages healthy beverage consumption, provides safe drinking water, and restricts SSB sales in cafeterias and vending machines can reduce SSB consumption among adolescents. In addition, there is a need for school- and community-based educational campaigns focusing on nutrition to increase awareness about the adverse effects of SSBs and propose healthy alternatives (e.g., water and milk without sugar) that can replace SSBs.

As parents have a significant influence on dietary habit formation (33), high education levels were associated with healthy dietary habit formation among adolescents (34, 35). According to Riediger et al. (34), individuals have opportunities to obtain health-related knowledge and learn health management methods. Thus, individuals' educational level might be associated with their level of health literacy (36); women tend to be more concerned about healthy diets than men (37). Furthermore, women take on the caregiver's role to improve their children's health (38). Thus, maternal health literacy based on educational level may influence SSB consumption. Considering these findings, nutritional education for improving maternal health literacy regarding SSBs can help reduce SSB consumption among adolescents.

In this study, current alcohol consumption, smoking, and decreased vegetable consumption were associated with increased SSB consumption among adolescent boys, whereas increased screen-based sedentary behavior was associated with increased SSB consumption among adolescent girls. Previous studies have also found that SSB consumption is accompanied by alcohol and smoking among adolescents (39, 40). High school boys in Korea have shown increased frequency and intensity of smoking and current and binge alcohol consumption compared to high school girls (15). According to Wiss et al. (41), alcohol consumption and smoking may induce sugar cravings by stimulating appetite through the biochemical mechanisms of neurotransmitters (e.g., dopamine, opioid peptides, and serotonin). Dinicolantonio et al. (42) proposed that sugar may have drug-like psychoactive effects, inducing dependence and/or addiction; that is, substance abuse and sugar are cross-sensitized, which may lead sugar to have a potential “gateway effect” on substance abuse (42). Considering the high prevalence of alcohol consumption and smoking among adolescent boys, there is a need for strategies to reduce alcohol and smoking consumption among adolescents.

Bjelland et al. (43) found that reduced vegetable consumption was closely associated with an increase in SSB consumption. A previous study found that boys showed a lower preference for vegetables as compared to girls, leading to less vegetable consumption in boys than in girls (44). This may imply that boys do not like the taste of vegetables that are less sweet, whereas girls tend to like the taste of various vegetables, which may influence their preference for vegetables. These sex differences were related to boys' perception of more barriers to vegetable intake than girls (45, 46). In addition, girls were more compliant with their parents' requests to eat vegetables, and they tried to regularly consume more vegetables for weight control and good health, regardless of their preferences (46, 47). Considering these findings, it is clear that adolescent boys who prefer sweet and tamed foods preferred strong SSBs. Thus, to reduce SSB consumption in adolescent boys, it is necessary to introduce healthy alternatives that are sweet.

Previous studies found that increased screen-based sedentary behavior was associated with increased consumption of energy-dense foods, including SSBs (19, 48). Furthermore, Sampasa-Kanyinga and Chaput (12) found that adherence to the recommended screen time (not more than 2 h a day) was negatively associated with SSB consumption among adolescent girls; however, there was no significant association between screen time and SSB consumption among adolescent boys. Thus, the researchers suggested that the association between screen-based sedentary behavior and SSB consumption may be sex-specific, showing a significant inverse association only among girls (12). According to Avery et al. (49), prolonged screen time (e.g., watching television) was associated with increased exposure to SSB advertisements. Thus, increased screen time was associated with greater SSB consumption and reduced fruit and vegetable consumption (50, 51). Women tend to spend more leisure time in the form of screen-based sedentary behaviors, such as watching television, whereas men may engage in physical activity during their leisure time. Furthermore, in Korea, the Confucian culture encourages girls to play games based on sedentary behaviors from an early age, whereas boys are encouraged to engage in active play based on physical activity. Thus, girls are more likely to spend their leisure time watching television (and engaging in screen-based sedentary behavior) as compared to boys. Therefore, girls may be exposed to more SSB advertisements as compared to boys. In light of these findings, reducing exposure to SSB advertisements by restricting screen-based sedentary behavior can be effective in reducing SSB consumption among adolescent girls.

This study may contribute to the development of sex-tailored interventions for reducing SSB consumption among adolescents. However, it has some limitations. First, SSB consumption was evaluated based on drinking days; however, we did not consider the frequency and amounts of SSBs consumed on drinking days. Second, owing to the limitations associated with secondary data analysis, some beverages containing sugar (e.g., milk and coffee) were not considered as SSBs in this study. Thus, the total amount of sugar intake from all types of SSBs was not calculated. Further studies must be conducted to determine the total amount of sugar intake by evaluating the frequency and amounts of all SSBs consumed by participants. Third, in considering potential associations, limited individual and environmental factors were included. Although the SSB consumption of adolescents was associated with their family environments (e.g., parental SSBs consumption, parenting styles toward SSB consumption, access to SSBs at home), family environmental factors were rarely involved in the environmental factors. Thus, in future studies, various family environmental factors should be considered as potentially associated environmental factors. Fourth, some variables (e.g., depressive symptoms, stress) were assessed using a single question, owing to the limitation of secondary data analysis. Thus, in future studies, structured instruments with good reliability and validity need to be used. Fifth, this study used a cross-sectional design. However, a longitudinal study may be more accurate in verifying the cause–effect relationships among independent and dependent variables. Finally, this study analyzed data only from high school students; however, individual and environmental factors associated with SSB consumption may differ for children and adolescents in elementary and middle schools. Thus, it is important for future studies to identify these factors for children and adolescents in elementary and middle schools.

## Conclusions

The current study identified sex-specific factors associated with SSB consumption in South Korean high school students. Current alcohol consumption, smoking, and reduced vegetable consumption were associated with increased SSB consumption among boys, whereas increased screen-based sedentary behavior was associated with increased SSB consumption among girls. Therefore, as a prerequisite for developing strategies for reducing SSB consumption, it is necessary to consider sex differences in the factors associated with SSB consumption in adolescents.

## Data Availability Statement

Publicly available datasets were analyzed in this study. The data set for this study is data from the 15th Korea Youth Risk Behavior Web-based Survey (2019) conducted Korea Disease Control and Prevention Agency and can be found online by following the academic research material application procedure; https://www.kdca.go.kr/yhs.

## Ethics Statement

Ethical review and approval was not required for the study on human participants in accordance with the local legislation and institutional requirements. Written informed consent to participate in this study was provided by the participants' legal guardian/next of kin. The current study was exempted from the Institutional Review Board's (IRB) review, as it was conducted using secondary data from the 15th KYRBS, 2019 (Approval no. 202109-SB-207-01).

## Author Contributions

JR conceptualized the article and analyzed the data. JR and MP contributed to the interpretation of the results and prepared the manuscript. All authors contributed to the article and approved the submitted version.

## Funding

This study was supported by a National Research Foundation of Korea (NRF) grant funded by the Korean government (Ministry of Science and ICT) (2021R1A2C100682811).

## Conflict of Interest

The authors declare that the research was conducted in the absence of any commercial or financial relationships that could be construed as a potential conflict of interest.

## Publisher's Note

All claims expressed in this article are solely those of the authors and do not necessarily represent those of their affiliated organizations, or those of the publisher, the editors and the reviewers. Any product that may be evaluated in this article, or claim that may be made by its manufacturer, is not guaranteed or endorsed by the publisher.
